# Derived high reducing sugar and lignin colloid particles from corn stover

**DOI:** 10.1186/s13065-020-00725-y

**Published:** 2020-12-10

**Authors:** Wei Liu, Shengnan Zhuo, Mengying Si, Mengting Yuan, Yan Shi

**Affiliations:** 1 School of Life Science, Tonghua Normal University, Tonghua, 134000 China; 2 School of Metallurgy and Environment, Central South University, Changsha, 410083 China; 3 Chinese National Engineering Research Center for Control & Treatment of Heavy Metal Pollution, Changsha, 410083 China; 4 School of Environment, Harbin Institute of Technology, Harbin, 150090 China

**Keywords:** Corn stover, THF–H_2_O pretreatment, Reducing sugar, Lignin, Lignin colloid particles

## Abstract

Lignocellulosic biomass is considered as the largest potential candidate to develop alternative energy, such as biofuel, biomaterial. However, the efficient conversion of cellulose and practical utilization of lignin are great challenges for sustainable biorefinery. In this study, high reducing sugar yield and different size of lignin colloid particles (LCPs) were obtained via tetrahydrofuran–water (THF–H_2_O) pretreatment of corn stover (CS). THF–H_2_O as a co-solvent, could efficiently dissolve lignin and retain cellulose. After the pretreatment, 640.87 mg/g of reducing sugar was produced, that was 6.66-fold higher than that of the untreated CS. Meanwhile, the pretreatment liquor could form spherical LCPs with different sizes ranged from 202 to 732 nm through self-assembly. We studied the optimal pretreatment condition to simultaneously realize the high reducing sugar yield (588.4 mg/g) and excellent LCPs preparation with average size of 243 nm was under TH22 (THF–H_2_O pretreatment at 120 °C for 2 h). To further explore the formation of LCPs with different sizes. We studied the lignin structure changes of various conditions, concluded the size of LCPs was related to the lignin concentration and syringyl/guaiacyl (S/G) ratio. As the increase of the lignin concentration and S/G, the sizes of LCPs were increased. G-type lignin was easier to dissolve in the mild pretreatment supernatant, contributing to form smaller LCPs with a good dispersibility. In the severe condition, both of S and G-type lignin were dissolved due to the lignin depolymerization, formed the larger sphere particles. This work provides a novel perspective for the technical design of lignocellulosic biomass conversion.

## Introduction

Lignocellulose, consisting of cellulose, hemicelluloses and lignin, is the most abundant biomass on earth [1]. 100 billion tons of lignocellulosic biomass are produced annually in the world, but less than 10% of them are utilized [2, 3]. However, the burning of biomass will cause air pollution and climate problems [4]. For the decades, significant progresses have been made for the conversion of lignocellulosic biomass to energy and material used in environment remediation [5, 6]. As the component with the highest content in lignocellulosic biomass, cellulose plays an important role in pulp and paper, fuel ethanol, and other industries [7, 8]. Compared with cellulose and lignin, the branched structure and lower degree of polymerization property of hemicellulose made it easier to be degraded when subjected to heat pretreatment [9]. Therefore, it is preferentially separated from lignocellulosic raw materials and converted into industrial products, such as functional sugar, furfural, and other chemicals [10]. Most of the refining schemes focus on utilizing convertible celluloses and hemicelluloses, leaving lignin behind in the selective conversion processes [11, 12]. Lignin, together with cellulose and hemicellulose, constitutes a complex structure of lignocellulosic biomass with cross-linking heterogeneity [13]. Traditionally, lignin extraction is easy to destroy the original structure of lignin and form more inactive C–C bonds, which is not conducive to the development and utilization of downstream industries [14]. Therefore, how to efficiently utilize lignin with low cost and aromatic nature to realize all components utilization has turned into a global conundrum in current biorefinery.

There is no doubt that achieving high content of sugar is the major goal in the advanced biorefinery process, for example, ethanol can be produced by the fermentation of sugars. In order to achieve the goal, removal of the outer lignin and retention of cellulose in lignocellulosic biomass has become the primary mission via pretreatment. Currently, a great many pretreatment strategies based on acid [15], alkali [16] and organic solvent [17] were employed to efficiently remove or solve lignin from lignocellulose, which improve the glucose releasing. Unfortunately, large amounts of lignin are wasted in the flushing way of pretreatment spent liquor. As we know, lignin as the natural aromatic compound with high carbon content, abundant bonds and functional groups, is a good precursor for the preparation of carbon-based materials including lignin fiber, activated carbon, carbon foam, and lignin nanomaterials [16, 18]. Therefore, the utilization of lignin liquor is necessary.

According to the recent literatures, we knew lignin based nanoparticles has gained increasing interest, with the potential application in the fields of medicine, catalysis, water treatment adsorbents, and antimicrobial agents [19]. Lignin nanoparticles preparation usually uses lignin from the paper and pulping industry or biorefinery process, which need further chemical modification. For example, the uniform colloidal spheres were prepared via self-assembly with alkali lignin recovered from the pulping black liquor and chemically modified by acetylating [20]. Currently, lignin nanoparticles directly prepared with lignin produced from lignocellulose pretreatment were obtained [21, 22], which not only avoids lignin modification, but also realizes the utilization of waste lignin. However, the preparation of lignin nanoparticles and enzymatic hydrolysis of carbohydrates are always regarded as the different and independent processes. For example, when we want to obtain the high sugar conversion, we would use the high concentration of acid or alkali or organic under high temperature to remove much more lignin, thus, lignin nanoparticles might not be prepared directly. Otherwise, low content of lignin removal can not meet the accessibility of cellulase to cellulose, reducing the sugar yield [23]. Therefore, designing a method to simultaneously realize the formation of lignin-based materials from pretreatment liquid and the conversion of cellulose to sugar is significant and meaningful to the biorefinery of biomass.

In our previous studies, a new strategy involved a synergy of lignocelluloses pretreatment by THF–H_2_O and bacterial strain to enhance enzymatic hydrolysis was developed [24, 25]. The result showed THF–H_2_O had a high lignin removal efficiency and retained a large amount of cellulose for sugar production. However, the pretreatment spent liquor was treated as wastewater after THF recovery. According to these literatures that lignin nanoparticles were prepared based on alkali lignin dissolved in THF [20, 26], we speculated the idea of synchronously achieve the high sugar yield and lignin nanoparticle using THF–H_2_O pretreatment is possible. It is worth noting that the shape and size of lignin nanoparticle were diverse. Many researchers reported the lignin nanoparticles with different shape and size were prepared from the different kinds of lignin (softwood kraft lignin, alkali lignin, Sigma-Aldrich kraft lignin) using different solvents (methanol, ethanol, DMF, THF) [26, 27]. It can be seen that, solvent, lignin type and experiment condition might be the effect factor on the particle size. Zhao also proposed that the solution structures of lignin is essential to prepare well-controlled lignin nanoparticles [28]. However, to date, though, as the above presented papers has proposed some causes of the particle size, little papers pay attention to the lignin nanoparticles formation combined with the biomass pretreatment and the role of lignin in the size variation of lignin particle.

In this work, the overall objective is to combine biomass pretreatment with lignin-based material preparation, we adopted the pretreatment pattern based on acid-catalyzed THF–H_2_O system, realizing the highly efficient enzymatic hydrolysis and formation of lignin colloid particles (LCPs) with appropriate size, elucidating the effect of lignin from pretreatment stream on the particle size. The specific objectives are: (1) study the enzymatic hydrolysis of CS under different pretreatment conditions (2) prepare the LCPs from the different pretreatment stream; and (3) elucidate the relationship between the lignin structure and the size of LCPs.

## Materials and methods

### Materials and chemicals

The corn stover (CS) was purchased from Jining, Shandong province, China. The CS after washing and drying was cut and crushed with a pulverizer, then sifted by 18 to 60-mesh griddles and air-dried for 24 h at 50 °C before use. Tetrahydrofuran (THF, A.R.) was purchased from a commercial Shanghai Macklin Biochemical Co., Ltd. Concentrated sulfuric acid (98 wt %) was purchased from ChengDu Chron Chemicals Co., Ltd. Other chemicals were purchased from a commercial source and used without any further purification. The whole research methodology was shown in Additional file 1: Figure S1.

### Biomass pretreatment

The CS was weighed at the ration of 1:20 (g/ml) with the THF/H_2_O (1:1) solution. The mixture was placed in a 100 ml of hydrothermal reactor. Then, the sulfuric acid (0.5wt %) was added as the catalyst. The hydrothermal reactor was put into the air drying oven after stirring. The temperature and time were 120 °C and 150 °C for 1 h, 2 h, and 4 h, respectively. The reactor was quickly cooled down by flushing the water after pretreatments. Then, the solid residues and pretreatment solution were separated by vacuum filtration. The solution was collected and stored in a refrigerator (4 °C). The solid residues were further filtrated by distilled water to remove THF until the pH was neutral, and then, dried at 50 °C for 24 h. The reaction condition was named as TH21, TH22, TH24, TH51, TH52, TH54, respectively.

#### Enzymatic hydrolysis and composition analysis

The enzymatic hydrolysis of dried THF–H_2_O pretreated CS residues (2.5% (w/v)) was performed with Cellulase Cellic (CTec2, Novozymes) and citrate buffer (0.05 M, pH 4.8) in the water bath shaker at 50 °C, 150 rpm for 72 h. In addition, the tetracycline (40 μg/ml) and cycloheximide (30 μg/ml) are added into the enzymatic hydrolysis system to prevent microbial contamination. And then, the supernatant was took out and centrifuged to measure the reducing sugar with the DNS method [29]. The reducing sugar yield was calculated by the following equation.

$${\text{Y}} = \frac{{{\text{c}} \times {\text{v}}}}{\text{m}}$$

where Y is the reducing sugar yield (mg/g), c is the sugar concentration (g/l), v is the system volume of enzymatic hydrolysis (ml), m is the mass of CS in the system of enzymatic hydrolysis (g).

All the experiments were done in triplicate. The composition including cellulose, hemicellulose, and lignin analysis was according to the previous literature [30]. All measurements were carried out in triplicate.

#### Weight loss analysis

The treated and untreated CS were washed three times, and dried in oven at 60 °C for 12 h. The weight was measured under dry condition. Then, the weight loss rate of CS was calculated with the different mass obtained before and after pretreatment [31]. All the experiments were done in triplicate.

#### Morphology analysis

The dried CS before and after pretreatments with different conditions were coated with gold and were detected with the scanning electron microscopy (SEM JSM-IT300LA, JEOL, Japan) [32].

### Preparation of LCPs

Pretreatment liquid from the biomass pretreatment under different conditions were transformed into different flasks. The excess of the deionized water was slowly added into each flask with constant magnetic stirring. Then, the colloid solutions containing LCPs from different pretreatment conditions were obtained as shown in Additional file 1: Figure S2. The prepared LCPs under various conditions was named as LCP21, LCP22, LCP24, LCP51, LCP52, LCP54, respectively.

### Characterization of LCPs

The morphology of LCPs was observed with the field emission scanning electron microscope (FESEM, FEI Nova NanoSEM230, Czekh). The size and dispersity of particles were analyzed using a Nano-ZS (Malvern Instruments, UK). The absorption peak of LCPs in aqueous solutions was measured using a U-4100 Spectrophotometer (Thermo Scientific Inc., USA). The purified lignin directly extracted from the pretreatment liquid was used as the positive control. The extracted lignin was dissolved in the solution of THF and water at the ratio of 1:1. The chemical groups of LCPs were confirmed via a Nicolet IS10 Fourier Transform Infrared spectrometer (FTIR, Thermo Fisher, USA).

### Extraction and characterization of lignin

In order to study the effect of lignin structure on the formation of LCPs, the lignin in the pretreatment liquid was extracted with the same method as the previous reported [30]. The extracted lignin samples were analyzed via a Bruker Avance 400 MHz two-dimensional heteronuclear single quantum coherence nuclear magnetic resonance spectrometer (2D-HSQC-NMR, Billerica, MA, USA) to identify the change in structural units of lignin structure and investigate the cleavage of linkage bonds [33]. The chemical groups of lignin were analyzed through the FTIR [34]. The Waters Alliance gel permeation chromatography 1515 (Waters, USA) was used to analyze the molecular weight of lignin [35]. Before the sample tasting, lignin was first acetylated with acetic anhydride and pyridine, then methanol to stop reaction, and nitrogen purging, vacuum drying. Herein, lignin extracted from various pretreatment liquid LCPs was named as lignin-TH21, lignin-TH22, lignin-TH24, lignin-TH51, lignin-TH52, and lignin-TH54.

## Results and discussion

### Efficient biomass pretreatment

#### The release of reducing sugar

**Fig. 1:**
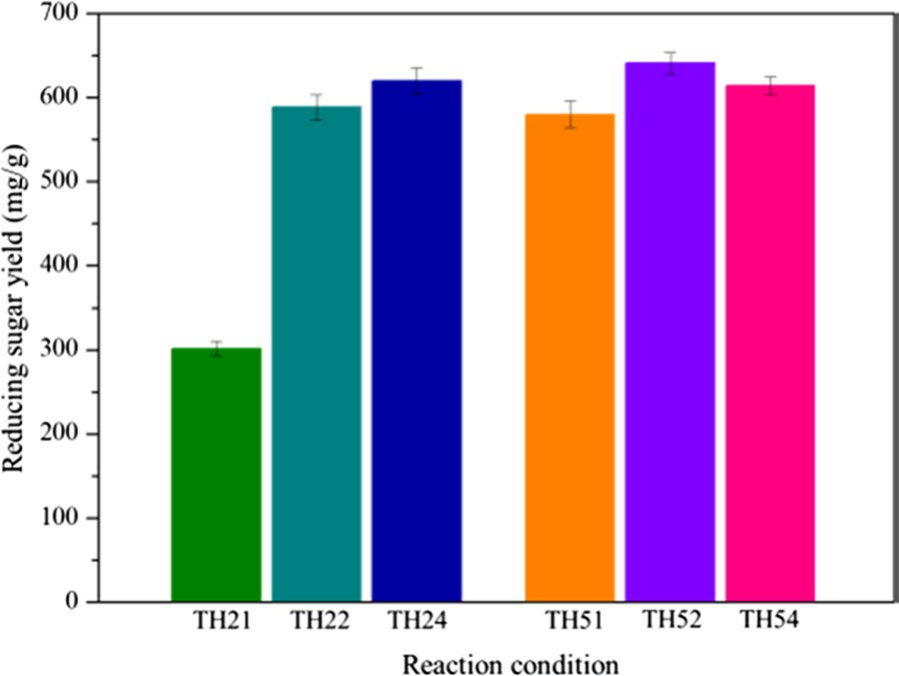
Reducing sugar yield of corn stover pretreatment under different conditions

Generally, pretreatment is the primary step for the utilization of biomass to sugars, ethanol or other compounds [36]. The sugar yields after enzymatic digestibility is the major criteria for determining the efficiency of pretreatments. Enzymatic hydrolysis of CS under all pretreatment conditions was carried out and the corresponding reducing sugar yield was investigated (Fig. 1). All the reducing sugar yields produced from CS that pretreated by THF-H_2_O system under different conditions were higher than that (84.7 mg/g) of the untreated [24]. They are 301.23 mg/g, 588.45 mg/g, 619.62 mg/g of TH21, TH22, TH24 and 579.83 mg/g, 640.87 mg/g and 613.9 mg/g of TH51, TH52, TH54. The maximum yield of 640.87 mg/g was obtained after pretreatment under TH52, which was highly competitive compared with others in previous works [15, 37]. In addition, the yield of the reducing sugar increased from 301.23 mg/g (TH21) to 619.62 mg/g (TH24) with the prolongation of reaction time under 120 °C, which was attributed to the breakdown of the biomass recalcitrance structure. A large amount of lignin was dissolved by THF–H_2_O, and the internal cellulose was gradually exposed, which promoted the accessibility of the cellulase to the substrate [25]. In general, the reducing sugar yield of biomass pretreated by THF–H_2_O increases with the increase of the pretreated temperature and time [25]. However, it was surprised to observe that the reducing sugar yield of CS pretreated at 150 °C increased from 579.83 mg/g (TH51) to 640.87 mg/g (TH52) first, and then decreased to 613.9 mg/g (TH54) with the increase of the pretreated time. Although enzymatic digestion was significantly improved due to lignin removal from CS by THF–H_2_O under low temperature, there is still lignin remained in the pretreated CS, may play a physical adsorption role of nonproductive binding with cellulase in the enzymatic hydrolysis [38, 39]. This lignin residue can be further dissolved and removed from cell wall structure under severe condition [40], which resulted that the reducing sugar yields under 150 °C are higher than that under 120 °C with similar pretreated times. Although most of lignin has been removed from CS under TH54, the reducing sugar yield was not increased compared to that of TH52. In general, the more lignin removed under severe condition, the more internal cellulose exposed to the outside, which was benefit to the enzymatic hydrolysis of cellulose. For this phenomenon, we speculated some of the exposed cellulose might be attacked, which resulted in a decrease in the amount of degradable cellulose. The conceivable proof will be discussed and proved depending on the SEM analysis.

#### Morphology change of CS

**Fig. 2:**
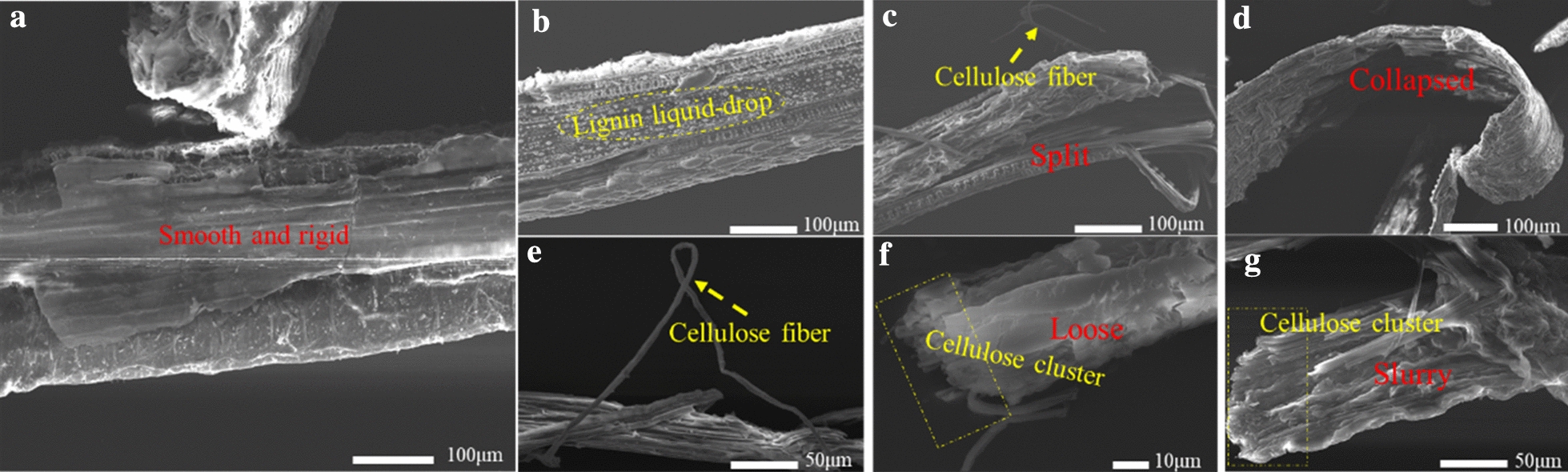
SEM of corn stover pretreatment under different condition (**a** un-treatment, **b** TH21, **c** TH22, **d** TH24, **e** TH51, **f** TH52, **g** TH54)

The purpose of pretreatment is not only to remove lignin on the surface of biomass as much as possible but also to maximize the retention of cellulose to obtain a high yield of the reducing sugar. The efficiency of biomass pretreatment is closely related to the change of its structure. To deep understand the reducing sugar production from pretreated CS, the morphology changes of CS before and after pretreatment were analyzed. The surface of the untreated CS (Fig. 2a) is smooth and rigid, covered with a large amount of lignin which is the physical barrier for cellulase [41]. In contrast, the surface of CS pretreated under TH21 was changed (Fig. 2b). Some lignin droplets were formed and deposited on the surface of the pretreated residues,which was similar to the phenomenon that was found in dilute sulphuric acid pretreatment of lignocellulosic biomass [15]. The formation of lignin droplets was caused by the acidic environment created by adding sulfuric acid as a catalyst in THF–H_2_O system. However, the lignin droplets remained on the surface of CS hindered the enzymatic hydrolysis, resulting in the low yield of the reducing sugar under TH21. In contrast, the morphology of CS pretreated under the other five reaction conditions has undergone tremendous changes. For example, the CS pretreated by TH22 was split and the cellulose fiber was observed (Fig. 2c). The CS was seriously deformed after TH24 pretreatment (Fig. 2d), indicating that the structure of the CS was fragile and collapsed [42]; No matter TH22 or TH24, the integrated structure of CS has been destroyed, the most importantly, cellulose appeared. It means that the enzymatic hydrolysis was easier than the CS covering with rigid lignin and lignin droplets. For TH51, CS was also cracked, showing the internal cellulose structure (Fig. 2e). Moreover, the fiber bundles can be seen in TH52 pretreated CS (Fig. 2f), which significantly increased the accessibility of cellulose to the enzyme, contributing to the highest reducing sugar yield. With the aggravation of reaction conditions, TH54 emerged a slurry structure (Fig. 2g), it was worth noting that although it retained the cellulose, its structure was not as loose as the TH52, which explained the cause of reducing sugar yield decrease.

#### Component change and mass loss of the CS

The change of CS structure after pretreatment will inevitably lead to the change of component and the loss of mass. Therefore, the analysis of component changes and mass loss before and after pretreatment was conducted.

The CS used in this study consists of 34.9% of cellulose, 38.5% of hemicellulose, 13.9% of lignin, and 12.7% of other non-organic and organic components [24]. As shown in Additional file 1: Figure S3, compared with the untreated CS, lignin and hemicellulose in the solid residual decreased from 13.87 to 10.02% and from 38.36 to 13.8%, respectively, with the increase of reaction time at 120 °C. It indicated that lignin and hemicellulose is continuously dissolved in THF–H_2_O system. The removal of lignin and hemicellulose also gradually increased at 150 °C. The content of lignin and hemicellulose in CS residue after pretreatment decreased from 12.8 to 6.59% and from 30.68 to 10.21%, respectively. Due to the removal of lignin, the content of cellulose was correspondingly increased. It changed from 47.71 (TH21) to 55.68% (TH24) and from 52.99 (TH51) to 71.71% (TH54), respectively. However, the highest cellulose content of 76.11% occurred in the TH52, which was best to the production of reducing sugar from TH52, the result was in line with the reducing sugar yield.

**Fig. 3:**
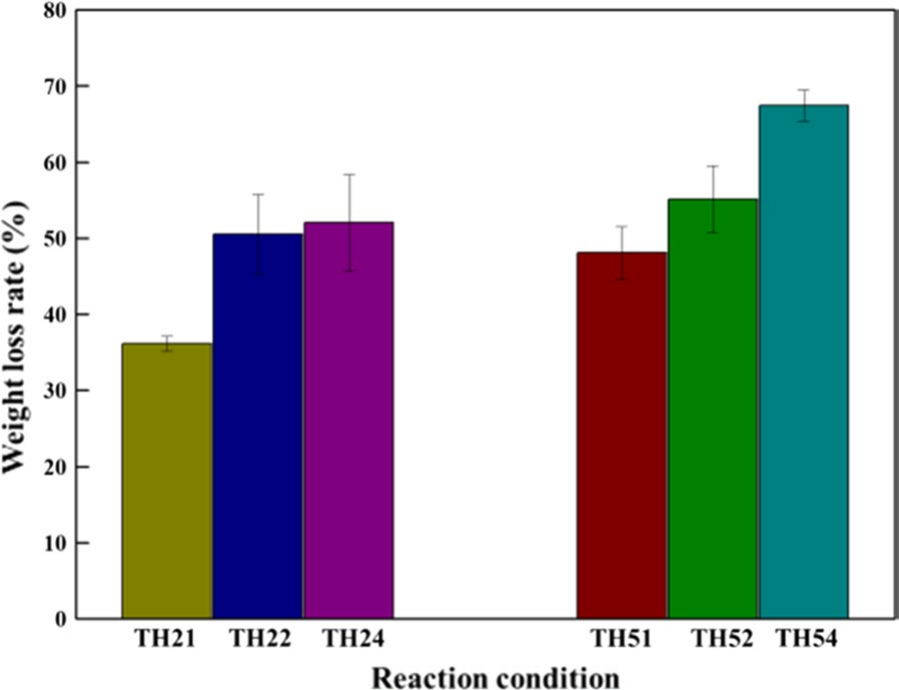
Weight loss of corn stover by the pretreatment under different conditions

The mass loss of treated CS was all increase from TH21 to TH24 and TH51 to TH54. The data were 36.14%, 50.54%, 52.00% and 48.11%, 55.11%, 67.46% under 120 °C and 150 °C, respectively (Fig. 3). The mass loss of CS under TH21 was the minimum of 36.14%. In addition, based on the analysis of the reducing sugar yield and the morphology of the CS, it can be inferred that the mass loss of CS is mainly attributed by the removal of a small amount of lignin and other components like hemicellulose by THF–H_2_O co-solvent system [42]. Most of the lignin formed droplets and deposited on the surface of the CS residue. Compared with TH21, the mass losses of TH22 and TH24 were markedly to 50.5% and 52% respectively, which were resulted from most of the hemicellulose and lignin removal (Additional file 1: Figure S3). Similarly, with the increase of reaction time, the more lignin was removed, the increasing mass loss was observed at 150 °C. Moreover, under TH54, some cellulose was degraded after lignin removal, which resulted in the mass loss reach to 67.46%, and it was unacceptable in the practical application.

Therefore, in order to maximize the yield of the reducing sugar from biomass, it is very important to optimize the pretreatment conditions. Compared with the other THF pretreatment [43, 44], in our work, the pretreatment method adopted the lower temperature (120 °C), and we also obtained the satisfying result. The most important reason we selected THF–H_2_O co-solvent as the pretreatment solvent is that the pretreatment liquid can be directly to prepare lignin nanoparticles [20, 26] rather than discarded.

### Formation of LCPs

The current utilization of biomass resources is involved in the utilization of both cellulose and lignin due to its unique structure. Therefore, in addition to the high reducing sugar yield obtained from CS by THF–H_2_O pretreatment, we directly used the pretreatment supernatant to prepare LCPs via introducing water into it without other chemical modification, which realized the efficient utilization of lignin in biomass. Different concentrations of colloid solution were formed based on the supernatants obtain from pretreatment under six different reaction conditions (Additional file 1: Figure S2). To understand the characteristics of the colloid solution, we adopted SEM to observe the morphology of LCPs, nano size analyzer to get information of particle size distribution statistics, Uv–vis to measure the concentration of colloid solution, and FTIR to analyze the functional groups of lignin particles.

#### Morphology and Size analysis of LCPs

**Fig. 4:**
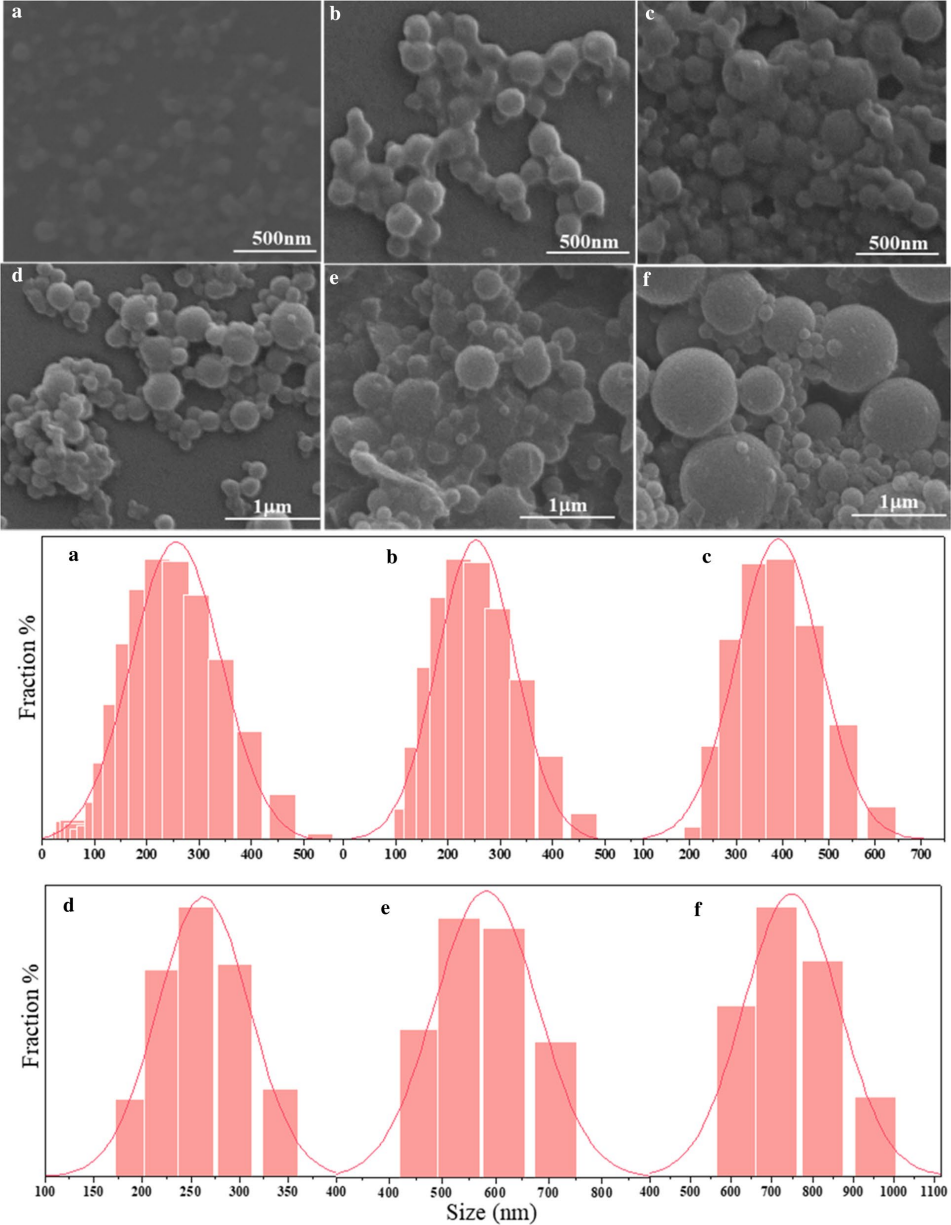
Size Distribution and FESEM of LCPs prepared from different pretreatment liquid. **a** LCP21, **b** LCP22, **c** LCP24, **d** LCP51, **e** LCP52, **f** LCP54

All the prepared LCPs were spherical and rich, concatenate (Fig. 4). The phenomenon is similar to other researches [27, 45], resulting from the electrostatic interaction. The particle size increased with the increase of the reaction time under the temperatures of 120 °C and 150 °C. LCP21 were very small and had a small number. The particle size mainly distributes between 164 and 295 nm, and the average particle size was 202 nm. The size distribution of LCP22 was 164–342 nm, which was a little bigger than that of LCP21. Its average particle size was 243 nm and the dispersion of particles was better than that of LCP21. LCP24 had a particle size distribution of 255–615 nm with the average particle size of 385 nm. Besides, it could be observed that the minimum particle size of LCP24 is larger than the average particle size of LCP21 and LCP22. Moreover, the particle sizes of LCP51, LCP52, and LCP54 were gradually increased. The corresponding particle sizes were 190–342, 459–712, 615–955 nm with the average particle sizes of 259, 547 and 732 nm, respectively. These results indicated that the lignin particle size gradually increased with the increase of reaction intensity. In addition, some particles of LCP54 even reached about ~ 1000 nm. The explanation for this result might be related to the lignin structure dissolved in different pretreatment solution. In addition, the LCPs prepared in this work was comparative to the other lignin particles with various shapes and sizes prepared by different methods (Additional file 1: Table S1).

To confirm the different particle size of LCPs was caused by the structure of lignin, we next adopted the UV wavelength scanning to observe the if the formation interaction changes.

#### Uv–vis analysis of LCPs

**Fig. 5:**
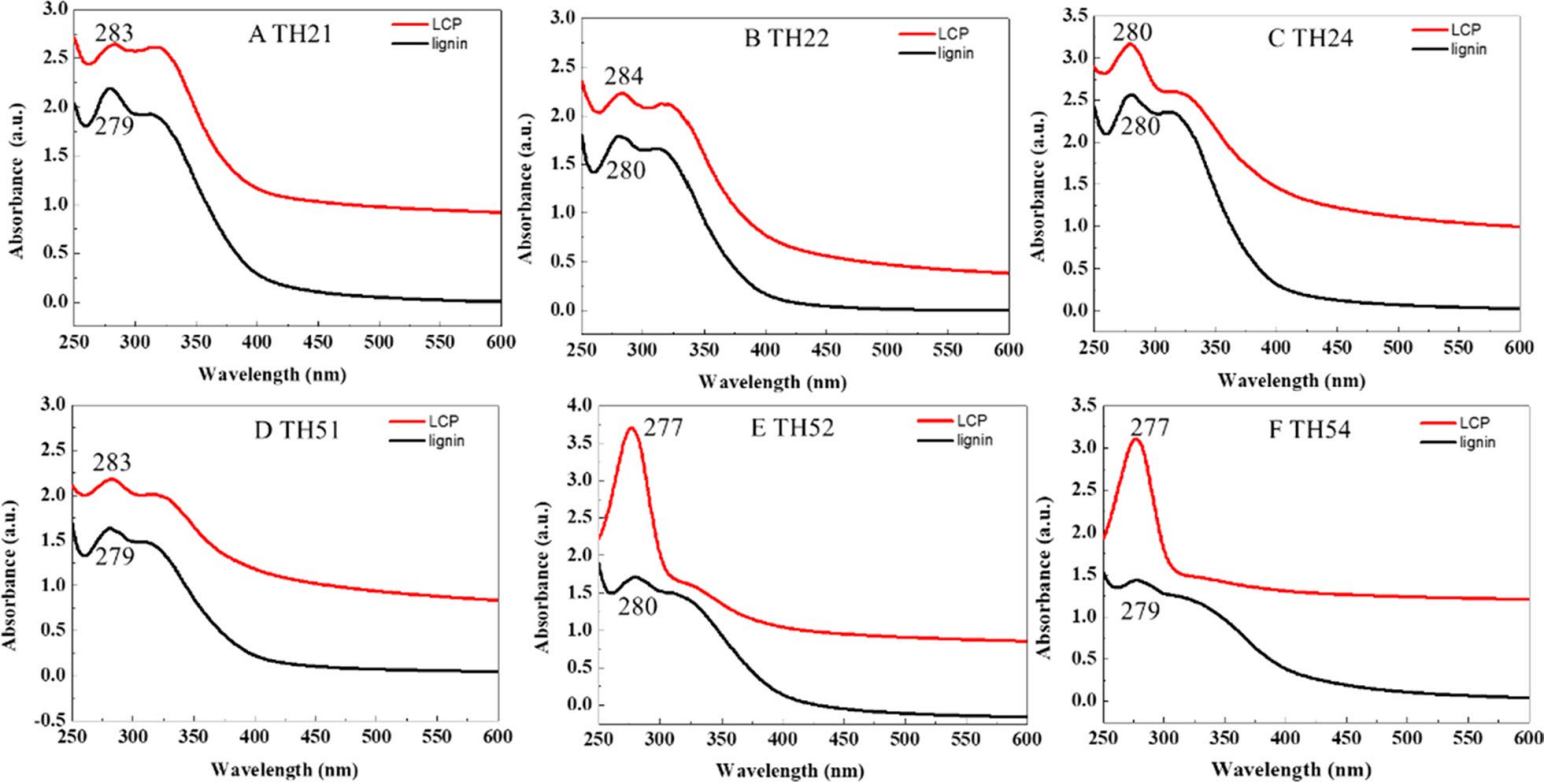
Uv–vis spectra comparison of lignin and LCPs from the pretreatment liquor under different conditions

Uv–vis was used to compare the lignin in the pretreatment solution with the corresponding LCPs to analyze the formation of LCPs. Previous studies have discussed that the LCPs are formed by the π–π interaction of orbitals of non-chemical bonds between aromatic rings in lignin molecule [20, 46]. Before and after the formation of colloid particles, the adsorption peak would shift under the π–π interaction [46]. As displayed in Fig. 5, the absorption peak of typical benzene compounds (~ 280 nm) was observed in the lignin and LCPs solution, suggesting the aromatic ring structure of lignin has not destroyed in the process of LCPs formation. It was consistent with the conclusion that π-π interaction in lignin caused the hydrophobic effect which brought about the formation of lignin colloid in solution [20]. The different shifts of maximum λ at ~ 280 nm indicated the weakening or strengthening of aromatic groups in π-stacking from lignin [20]. Lignin consist of three units including guaiacyl, syringyl, and phydroxyphenyl, their aromatic structure is different [19]. Therefore, the variation in π-stacking may be attributed to the different aromatic structures of lignin dissolved in the pretreatment liquid. Thus, we will conduct the effect research of lignin structure on LCPs.

Moreover, we found the LCP24, LCP52, and LCP54 had a higher absorption peak at maximum λ, which were related to their high concentration that can be seen in Additional file 1: Figure S2. The higher concentration of LCPs depended on the increase in lignin dissolution with the increase of pretreatment reaction degree.

#### FTIR analysis of LCPs

**Fig. 6:**
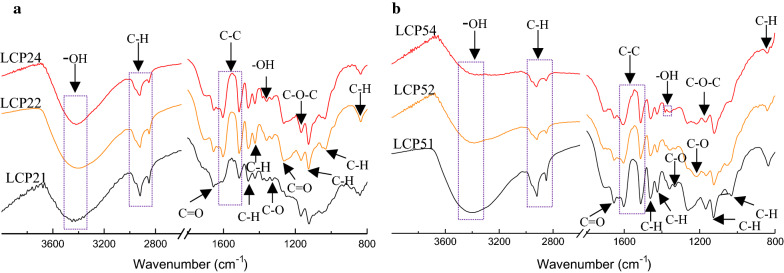
FTIR spectra of LCPs prepared from different pretreatment liquid

The structural properties of materials determine their application values. Therefore, FTIR was used to characterize the functional groups of LCPs. As shown in Fig. 6, the abundant functional groups were visually observed in the LCPs prepared from pretreatment solutions at 120 °C and 150 °C. The broad absorption peak at ~ 3400 cm^−1^ and the band at ~ 1379 cm^−1^ represent –OH stretching of phenolic and aliphatic moieties. The bands at 2918 cm^−1^ and 2849 cm^−1^ represent C-H stretching [47]. Aromatic skeleton stretching occurs at ~ 1600 cm^−1^, ~ 1515 cm^−1^, ~ 1426 cm^−1^. The C=O stretching in guaiacyl (G) unit appeared at ∼ 1267 cm^−1^ [48]. All these location peaks suggested that there is no significant change in the aromatic structure of lignin in the process of LCPs formation; and the particle surface modified with high phenolic hydroxyl and carboxyl functional groups is good for the solubility and stability of LCPs. In addition, LCPs have extra function groups of C=O (at ~ 1330 cm^−1^ and ~ 1265 cm^−1^) and C–O–C (at ~ 1165 cm^−1^) compared with the lignin [49]. In recent years, the LCPs with plenty of functional groups have been reported to play an important role in the adsorption materials applied in the environment field and other composite materials preparation for biomedical or cosmetic fields [18, 50]. Therefore, the LCPs in our study have the potential to be applied in these fields.

### Influence of lignin on the LCPs

Spherical LCPs with different sizes were prepared in different pretreatment solution. The formation of LCPs is closely related to the structure and concentration of dissolved lignin [28], therefore, we not only performed component analysis, but also extracted the lignin from the liquid and analyzed their molecular weight and structural changes by GPC, FTIR, and 2D-HSQC-NMR to elucidate the impact mechanism of lignin on the formation of LCPs.

#### GPC analysis

The analysis of the changes in lignin molecular weight through GPC can be used to characterize the degree of lignin polymerization after THF–H_2_O pretreatment. The deconstruction of the macromolecular lignin can be characterized via three indexes including weight-average molecular weight (M_w_), number-average molecular weight (M_n_), and dispersive poly-dispersity (PDI).

**Fig. 7:**
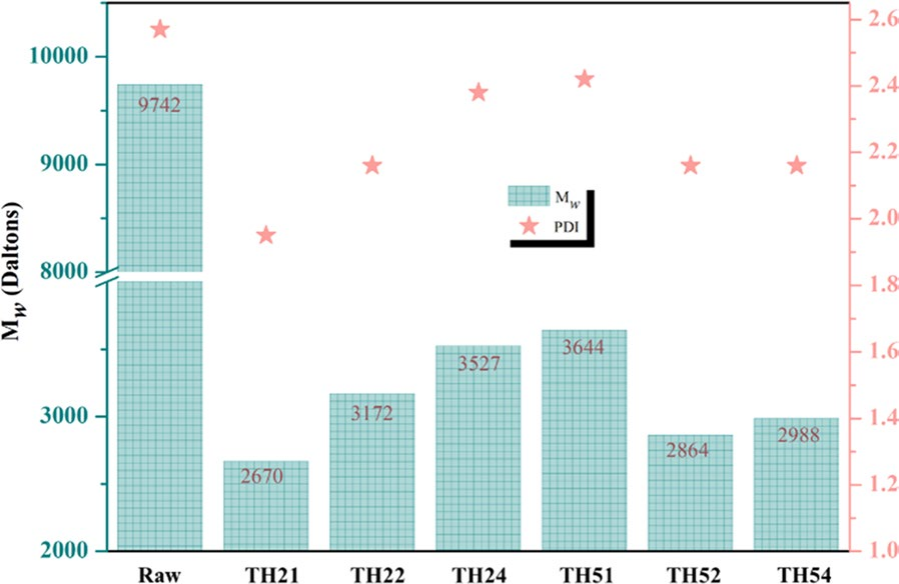
M_*w*_ and PDI of lignin extracted from different pretreatments

Figure 7 displayed the M_w_ and PDI of lignin extracted from different pretreatment solutions. The lignin extracted from the raw CS had the highest M_w_ (9742 Da) and PDI (2.57), indicating the high polymerizability and complexity of lignin in CS [51]. Compared with the raw lignin, the M_w_ of lignins in the pretreatment solution drastically decreased. The M_w_ and PDI increased from 2670 and 1.95 of lignin-TH21 to 3527 and 2.38 of lignin-TH24, respectively. In addition, the highest M_w_ (3644) and PDI (2.42) were obtained in lignin-TH51, then, decrease to 2988 and 2.16 of lignin-TH54. Combined with the above “The release of reducing sugar” section analysis, it can be inferred that lignin with small molecule weight was initially dissolved by THF–H_2_O under TH21, and the molecular weight distribution (MWD) is narrow; Then, more and more lignin with large molecule weight was dissolved, the corresponding MWD gradually widened. The molecule weight of dissolved lignin from TH51 reached to the maximum. Next, the dissolved lignin started to depolymerize as the reaction condition continued to intensify. However, some of depolymerized lignin might repolymerize under severe condition [52, 53]. Therefore, the M_w_ of lignin-TH54 was a little higher than that from lignin-TH52. It seems to explain the increased size of LCPs was because of the increase of M_w_ from lignin-TH21 to lignin-TH24. However, it was not suitable to the trend from LCP-TH51 to LCP-TH54. Thus, it is necessary to further analyze the specific changes of lignin structure and ensure the form of lignin units in THF–H_2_O.

#### FTIR analysis

Based on the reducing sugar yield and the characteristics of the prepared LCPs, the lignin-TH22 and lignin-TH52 were selected for FTIR and 2D-HSQC-NMR analysis to further investigate the effect of lignin structure on the size of LCPs.

**Fig. 8:**
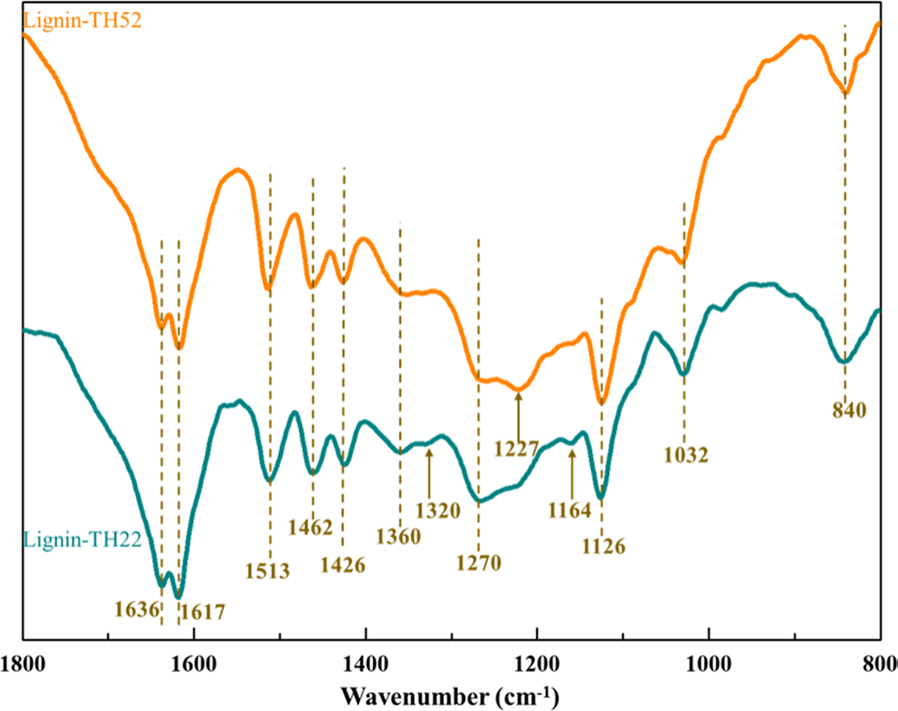
FTIR spectra of lignin extracted from TH22 and TH52

Obviously, FTIR spectra (Fig. 8) showed that most of the characteristic peaks of lignin-TH22 and lignin-TH52 still remained in these two samples [54, 55]. The peaks at 1617 cm^−1^, 1513 cm^−1^, and 1426 cm^−1^ were related to the vibration of aromatic ring skeleton, and the peak at 1462 cm^−1^ represented the C-H deformation of aromatic ring skeleton. These peaks indicated the existence of benzene structure in lignin-TH22 and lignin-TH52 [24]. However, compared with lignin-TH22, the peak at 1320 cm^−1^ which represented C=O stretching in syringyl (S) derivatives disappeared in lignin-TH52. In addition, the G ring breathing with C=O stretching (at 1270 cm^−1^) significantly decrease from lignin-TH52 [56]. Moreover, the peak at 1164 cm^−1^ which was attributed to the C–O–C vibration only remained in lignin-TH22. The peak at 1227 cm^−1^ associated with C–C, C–O, and C=O stretching only appeared in lignin-TH52. These changes might be caused by the break of linkage bonds due to the severe pretreatment. The destruction of linkages resulted in the changes of lignin structure units (i.e. S and G), which might be the key factors to explain the formation of LCPs with different size. In order to further confirm the speculation, we adopted the 2D-HSQC-NMR analysis to illustrate the structural changes of lignin.

#### 2D-HSQC-NMR analysis

**Fig. 9:**
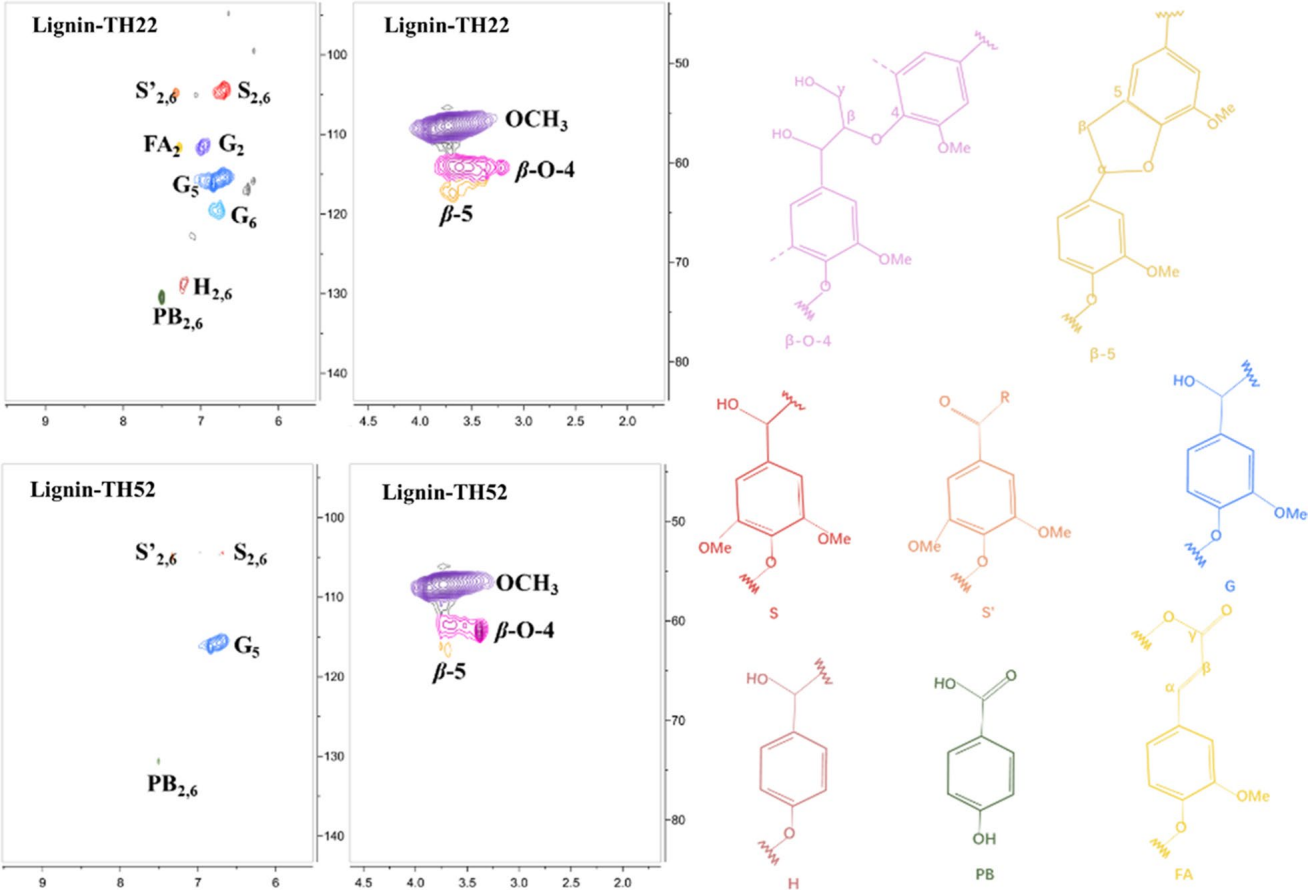
Aromatic region and side-chain in the HSQC-2DNMR spectra of lignins. S: syringyl units, S’: oxidized syringyl units, G: guaiacyl units, H: p-hydroxyphenyl units, PB: p-hydroxybenzoate substructures, FA: ferulates

Generally, the lignin consists of three basic units (S, G and p-hydroxyphenyl (H)) and linked by *β*-O-4, *β*-*β, β*-5 etc. [57]. All these signals can be reflected through the 2D-NMR spectra. The signal information of side chain region (δ_C_/δ_H_ 50–90/2.5–6.0) and aromatic region (δ_C_/δ_H_ 100–135/5.5–8.5) of lignin-TH22 and lignin-TH52 was displayed in Fig. 9. And the main cross-signals of lignin related to HSQC spectra were listed in Additional file 1: Table S2.

In the side chain region, the signals of *β*-O-4 and *β*-5 bonds in lignin-TH22 is stronger than that of lignin-TH52, indicating the lignin in the dissolved process was severely destroyed under TH52. In the aromatic region, the S-type and G-type of lignin-TH52 significantly decreased, and the signals of H-type lignin completely disappeared. In contrast, the signals of all three basic lignin units (S, G and H-type) remained in lignin-TH22. Specifically, the G-type lignin-TH22 were much higher than that of lignin-TH52. It is suggested that THF–H_2_O can dissolve the three types of lignin initially, and H lignin was not destroyed in lignin-TH22. However, the portion of G-type lignin was depolymerized in lignin-TH52. It is worth noting that it is easier for high content of G-type or low S/G lignin to form small size LCPs [21]. Therefore, in order to achieve the preparation of LCPs with smaller particle size, the reaction solvent must have the ability to dissolve more G-type lignin or control the deconstruction of G-type lignin via the suitable reaction environment. In our work, the size of LCP22 was lower than that of LCP52, suggesting the THF–H_2_O solvent under TH22 efficiently dissolved more types of lignin, the G-type lignin under TH52 was not as much as TH22 due to its depolymerization.

#### The concentration of lignin in the supernatant

The concentration of lignin depends on the amount of lignin removed after pretreatment. Lignin removal was increased with the increase of the reaction intensity, what was demonstrated by the component change (Additional file 1: Figure S3). Component analysis showed that the order of lignin content removed from the CS was TH54 > TH52 > TH24 > TH52 > TH22 > TH21. The highest concentration was obtained under TH54. Besides, lignin concentration could be directly observed from the color and turbidity of the LCPs solution (Additional file 1: Figure S2). Size analysis of LCPs indicated that the change of particle size is consistent with the change of lignin concentration. And the largest size of LCPs was obtained under TH54, which may be because the gather between particles. At the same volume, the particle size in the solution with low concentration of lignin is smaller due to the less content of lignin.

**Fig. 10:**
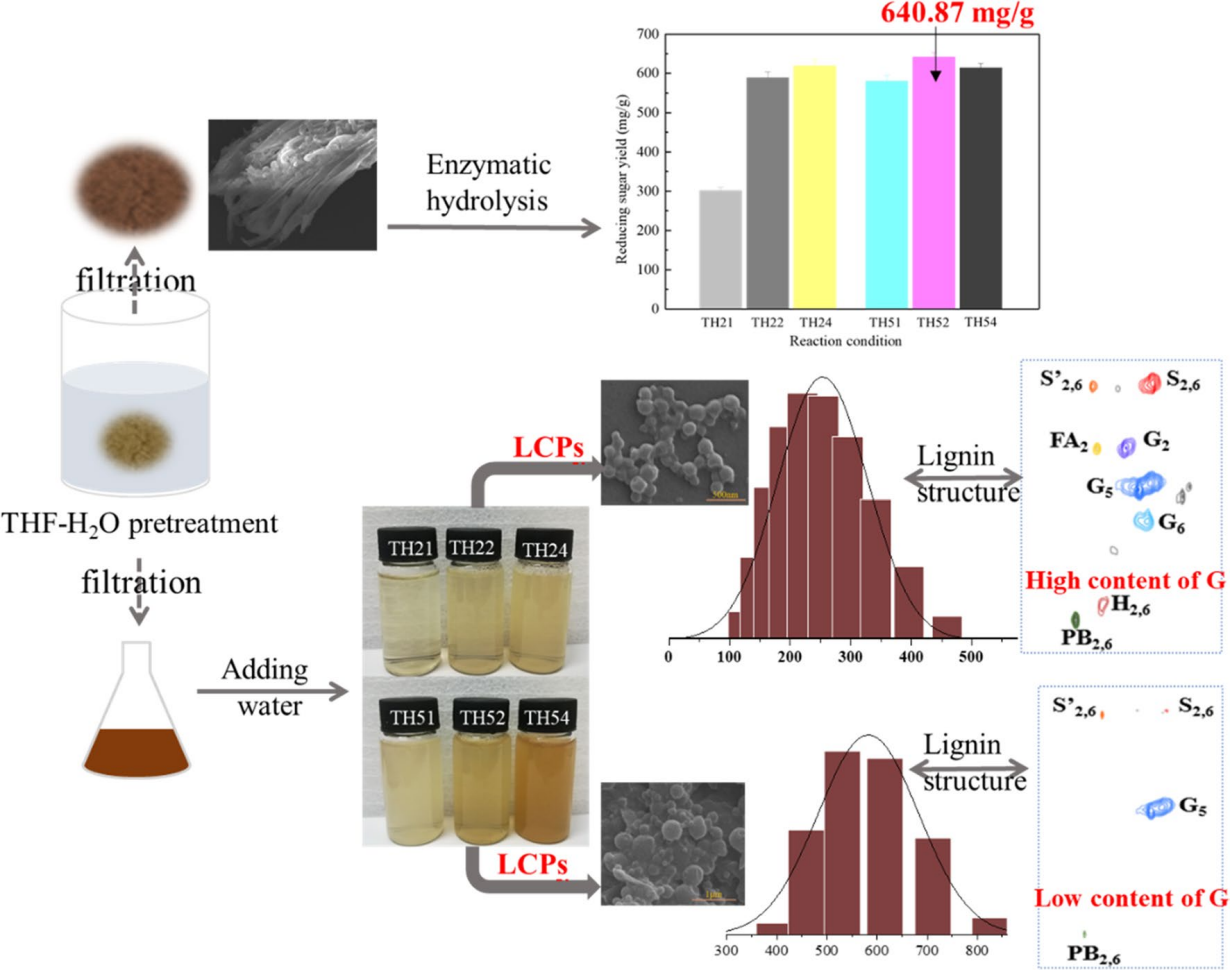
A schematic process flow diagram illustrating the high reducing sugar production and lignin colloid particles formation from CS by THF–H_2_O pretreatment

Lignin from the pretreatment solution under TH52 cannot form good LCPs even if the maximum yield of reducing sugar was obtained. However, pretreatment under TH22 realize the preparation of LCPs with good properties and high reducing sugar yield. Therefore, the pretreatment under TH22 was more practical in the actual biorefinery. The schematic of the sugar conversion and the mechanism of the formation of LCPs was fabricated as shown in Fig. 10. This strategy provides a practical guiding significance for the design of biomass pretreatment and the preparation of LCPs.

## Conclusions

In this study, efficient reducing sugar and lignin colloid particles were simultaneously obtained based on the CS pretreatment by THF–H_2_O with the acid catalytic system. Under the optimum pretreatment condition of TH22, a high reducing sugar yield of 588.45 mg/g and LCPs with good dispersibility and average particle size of 243 nm were obtained. Lignin removal and structure destruction of CS promoted the conversion of cellulose to sugar yield. More G-type lignin was dissolved in THF–H_2_O solution under TH22, as the basic solution to form the LCPs with appropriate sizes. In contrast, due to the depolymerization of lignin under the severe condition, leading to the decrease of G-type content or the increase of S/G, which resulted in the formation of larger particle that limited its application in future. Our work provides an advanced design idea for biorefinery in material preparation and energy utilization through the elucidation of influence of lignin structure on lignin-based material formation. It was meaningful to the development of cellulose conversion and the utilization of lignin. In the future, we will pay attention to the application of LCPs, hoping to discover its application potential in environment, medicine, biology and so on.

## Supplementary information

**Additional file 1: Figure S1.** Flowchart of the whole study process. **Figure S2.** Lignin colloid solution from different pretreatment condition. **Figure S3.** Chemical composition of corn stover before and after the pretreatment. **Table S1.** The lignin nanoparticles prepared from various methods. **Table S2.** Assignment of Main Lignin ^13^C-^1^H Cross-Signals in the HSQC spectra of the lignin fractions.

## Data Availability

All data generated or analyzed during this study are included in this published article.

## Abbreviations

CS: Corn stover
THF–H_2_O: Tetrahydrofuran–water
G: Guaiacyl
S: Syringyl
H: *p*-Hydroxyphenyl
LCPs: Lignin colloid particles
TH21: 120 °C for 1 h
TH22: 120 °C for 2 h
TH24: 120 °C for 4 h
TH51: 150 °C for 1 h
TH52: 150 °C for 2 h
TH54: 150 °C for 4 h
LCPxx: Colloid particles prepared from THxx pretreatmentnumber-average molecular weight
PDI: Poly-dispersity
Mw: Weight-average molecular weight
Mn: Number-average molecular weight
MWD: Molecular weight distribution
FESEM: Field emission scanning electron microscope
FTIR: Fourier Transform Infrared spectrometer
Uv–vis: Ultraviolet visible spectroscopy
GPC: Gel permeation chromatography
HSQC-NMR: Heteronuclear single quantum coherence nuclear magnetic resonance
