# Supplementary material for: Derived high reducing sugar and lignin colloid particles from corn stover
Source: BMC Chem. 2020 Dec 10;14(1):72. doi: 10.1186/s13065-020-00725-y (PMC7727252; doi:10.1186/s13065-020-00725-y)
Supplement: Supplementary file 1 — Additional file 1: Figure S1. Flowchart of the whole study process. Figure S2. Lignin colloid solution from different pretreatment condition. Figure S3. Chemical composition of corn stover before and after the pretreatment. Table S1. The lignin nanoparticles prepared from various methods. Table S2. Assignment of Main Lignin 13C-1H Cross-Signals in the HSQC spectra of the lignin fractions. [file 13065_2020_725_MOESM1_ESM.docx]

# Supporting Information

**Derived high reducing sugar and lignin colloid particles from corn stover**

Wei Liu ^a^, Shengnan Zhuo^* b, c, d^, Mengying Si ^b, c^, MengTing Yuan^b, c^, Yan Shi ^b, c^

*^a^* School of life Science, Tonghua Normal University, Tonghua, 134000, China.

*^b^* School of Metallurgy and Environment, Central South University, Changsha 410083, China

*^c^* Chinese National Engineering Research Center for Control & Treatment of Heavy Metal Pollution, Changsha 410083, China.

*^d^* School of Environment, Harbin Institute of Technology, Harbin 150090, China.

* Corresponding author: [zhsnan@126.com](mailto:zhsnan@126.com)


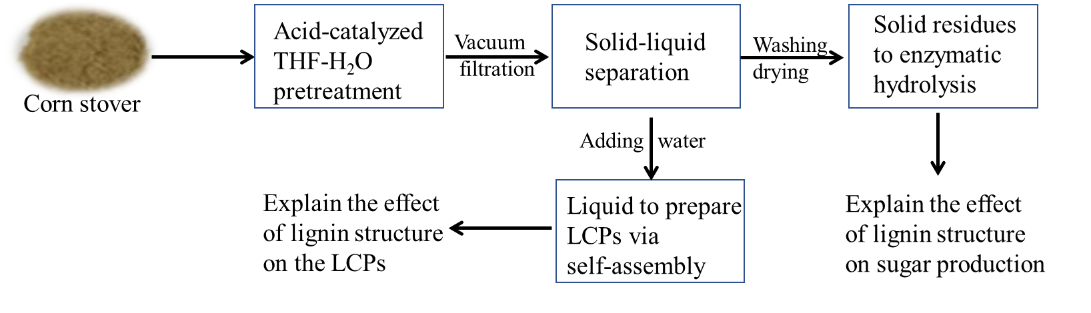


Figure S1. Schematic diagram for research methodology


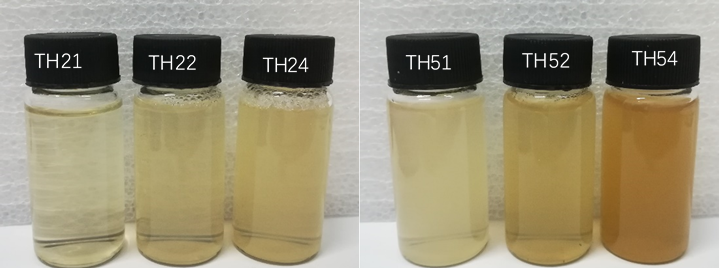
Figure S2. Lignin colloid solution from different pretreatment condition

Figure S3. Chemical composition of corn stover before and after the pretreatment

Table S1 The lignin nanoparticles prepared from various methods

| Morphology | Preparation  method | Size (nm) | Shape | Reference |
| --- | --- | --- | --- | --- |
| Nanoparticles | Solvent extraction | 218 | Sphere-likely | [[1](#_ENREF_1)] |
| Nanoparticles | Self-assembly | 190-590 | Sphere | [[2](#_ENREF_2)] |
| Nanoparticles | Anti-solvent  precipitation | 154–762 | - | [[3](#_ENREF_3)] |
| Nanoparticles | Precipitation | 50-500 | - | [[4](#_ENREF_4)] |
| Nanoparticles | Self-assembly | 200–500 | Spheres | [[5](#_ENREF_5)] |
| Nanoparticles | Self-assembly | 202–732 | Spheres | This study |

Table S2 Assignment of Main Lignin ^13^C-^1^H Cross-Signals in the HSQC spectra of the lignin fractions [[6](#_ENREF_6)]

| Lable | δ_C_/δ_H_ | Assignment |
| --- | --- | --- |
| -OCH_3_ | 55.9/3.73 | C-H in methoxyls |
| β-O-4 | 59.5-59.7/3.41-3.63 | C-H in β-O-4 substructures |
| β-5 | 62.5/3.73 | C-H in phenylcoumaran substructures |
| S_2,6_ | 103.8/6.71 | C_2,6_-H_2,6_ in etherified syringyl units |
| S’ _2,6_ | 106.2/7.27 | C_2,6_-H_2,6_ in oxidized syringyl units |
| G_2_ | 110.9/6.98 | C_2_-H_2_ in guaiacyl units |
| G_5_ | 114.9/6.71 | C_2_-H_2_ in guaiacyl units |
| G_6_ | 119/6.79 | C_6_-H_6_ in guaiacyl units |
| H_2,6_ | 127.8/7.19 | C_2,6_-H_2,6_ in p-hydroxyphenyl units |
| PB_2,6_ | 131.3/7.82 | C_2,6_-H_2,6_ in p-hydroxybenzoate substructures |
| FA_2_ | 112.0/7.22 | C_2_-H_2_ in ferulates |

References:

1. Myint, A.A.; Lee, H.W.; Seo, B.; Son, W.; Yoon, J.; Yoon, T.J.; Park, H.J.; Yu, J.; Yoon, J.; Lee, Y. One pot synthesis of environmentally friendly lignin nanoparticles with compressed liquid carbon dioxide as an antisolvent. *Green Chem*. 2016, 18, 2129-2146.

2. Lu, Q.; Zhu, M.; Zu, Y.; Liu, W.; Yang, L.; Zhang, Y.; Zhao, X.; Zhang, X.; Zhang, X.; Li, W. Comparative antioxidant activity of nanoscale lignin prepared by a supercritical antisolvent (SAS) process with non-nanoscale lignin. *Food Chem*. 2012, 135, 63-67.

3. Gupta, A.K.; Mohanty, S.; Nayak, S. Influence of addition of vapor grown carbon fibers on mechanical, thermal and biodegradation properties of lignin nanoparticle filled bio-poly(trimethylene terephthalate) hybrid nanocomposites. *RSC Advances*. 2015, 5, 56028-56036.

4. Gilca, I.A.; Popa, V.I.; Crestini, C. Obtaining lignin nanoparticles by sonication. *Ultrason Sonochem*. 2015, 23, 369-375.

5. Lievonen, M.; Valle, J.; Mattinen, M.; Hult, E.; Lintinen, K.; Kostiainen, M.A.; Paananen, A.; Szilvay, G,R.; Setälä, H.; Österberg, M. A simple process for lignin nanoparticle preparation. *Green Chem*. 2016, 18, 1416-1422.

6. Yuan, T.Q.; Sun, S.N.; Xu, F.; Sun, R.C. Structural characterization of lignin from triploid of *Populus tomentosa* Carr. *J Agric Food Chem*. 2011, 59, 6605-6615.
